# Expectations About Satiety and Thirst Are Modified by Acute Motivational State

**DOI:** 10.3389/fpsyg.2018.02559

**Published:** 2018-12-11

**Authors:** Martin R. Yeomans, Lucy Chambers, Keri McCrickerd

**Affiliations:** ^1^School of Psychology, University of Sussex, Brighton, United Kingdom; ^2^British Nutrition Foundation, London, United Kingdom; ^3^Clinical Nutrition Research Centre, Singapore Institute for Clinical Sciences, Agency for Science, Technology and Research (A^∗^STAR), Singapore, Singapore; ^4^National University Health System, Singapore, Singapore

**Keywords:** satiety, thirst, hunger, expectations, motivational state

## Abstract

Prior research has shown that consumers have clear and measurable expectations about the likely effects of food and drink items on their appetite and thirst, which are acquired with experience and influenced by a product’s taste and texture. What is unclear is whether expression of these expectations also varies with current appetitive state. It is possible that current appetite could increase or decrease the relevance of these expectations for future food choice and magnify a product’s expected impact on appetite. To test this, we contrasted expectations about satiety and thirst for four products consumed 2 h after an appetite manipulation at breakfast, achieved through *ad libitum* access to low-energy drinks only (hunger condition), cereal only but no drinks (thirst condition) or both foods and drinks (sated condition). The test products were two soups and two drinks, with a thicker and thinner version of each product type to act as positive control to ensure sensitivity in detecting differences in expectations. For satiety, the predicted differences between products were seen: soups and thicker products were expected to be more filling and to suppress subsequent hunger more than drinks and thinner products, but these differences were more pronounced in the hunger than thirsty or sated conditions. Being thirsty also enhanced expectations of how much drinks would appease immediate thirst. Overall the data show that expectations were adjusted subtly by a person’s current appetitive state, suggesting that we have mechanisms that highlight the most important features of a product at the time when it may be most beneficial to the consumer.

## Introduction

Consumers have a variety of expectations about food and drink products prior to consumption, which are known to influence product choice (Cadotte et al., 1987; Creusen and Schoormans, 2005). The degree to which any product then meets these expectations is known to impact overall consumer satisfaction, both for food and drink products (Cardello et al., 1985; Cardello, 2007) and more generally (Giese and Cote, 2000). The majority of research on expectations has focussed on those relating to the expected sensory characteristics of products and how these impact liking (Deliza and Macfie, 1996; Piqueras-Fiszman and Spence, 2015). In the case of food and drink products, another key class of expectations relate to the degree to which consumers anticipate a product will impact their appetite and thirst, such as whether they are expected to be filling (expected satiation) or to suppress hunger post consumption (expected satiety: Brunstrom, 2011).

Studies shows that consumers have measurable and consistent expectations of satiation and satiety, which vary between products and relate to some degree to their nutrient content and the sensory characteristics (Brunstrom et al., 2008; Ferriday et al., 2013; Forde et al., 2013, 2017; McCrickerd et al., 2015). However, these studies also show considerable differences in expected satiety between products with the same caloric density, demonstrating that expected satiety is not a simple proxy rating of actual energy content. The current view is that expected satiation and satiety are underpinned by past experience of the impact of the ingested food on appetite (Brunstrom, 2011), particularly when that food has previously been eaten to fullness (Irvine et al., 2013), and that humans appear better at discriminating lower energy dense foods based on their expected satiating power (Brunstrom et al., 2018). For this reason, foods typically consumed as meals are rated as having higher expected satiety (calorie-for-calorie) than those that are consumed as snacks, since the latter tend to be high energy density and rarely eaten to satiety even though snack foods typically have high energy density. What is untested is the extent to which the expression of these largely learned expectations of satiety depend on the appetite state at the time when the product is experienced. Indeed, in most studies of expected satiation and satiety to date, hunger state at the time of the study was uncontrolled. The overall aim of the study reported here was, therefore, to test whether expectations about how satiating the same product would be varied with current motivational state. Theoretically, if expected satiety is the cued recall of the likely satiating effects of a product when it is next encountered, then these memories should be independent of acute hunger since they are a product of the memory from previous experiences of consuming the to-be-ingested item. However, memory for food items is multi-faceted: we can recall different aspects of a product such as its expected sensory characteristics, the likelihood that we will experience pleasure ingesting it as well as the anticipated impact on appetite and thirst. An alternative theoretical position on the possible effects of hunger state on expected satiety is that we attend more to the likely satiating effects of a food when we are ourselves hungry, and less when not hungry. If so, then differences between products in the extent to which they are expected to be filling and reduce our appetite may be greater when we make the evaluation hungry than when sated. Such an effect would make sense in relation to appetite control since it would help consumers focus on features of a product most likely to satisfy their current need state. To our knowledge no previous study has explored this possibility.

Along with expectations about effects of products on appetite, liquid products are consumed either in response to, or anticipation of, thirst (Saltmarsh, 2001; Thornton, 2013). Whereas expectations about satiety have been widely explored, expectations relating to differences in the degree to which different drinks are expected to quench or suppress thirst remain relatively unexplored. Most studies on expectations about thirst have focussed on the sensory characteristics that are more or less associated with thirst quenching. For example, using a questionnaire measure it was reported that clear or brown colored fruit punch drinks were expected to reduce thirst more than would similar drinks with other colors (Clydesdale et al., 1992), while others reported that clear liquids were expected to be most thirst quenching (Zellner and Durlach, 2002, 2003). Together, these studies demonstrate that consumers readily make relative judgments on the likely impact of beverages on thirst (thirst expectations) as well as impacts on appetite (expected satiety), a point first made some years ago (French et al., 1993). A recent study further highlighted how consumers can readily evaluate satiety and thirst expectations for commonly consumed products (McCrickerd et al., 2015), and utilize different cues for each expectation: expected satiety was associated with overall energy content and sensory cues such as creaminess, whereas expected thirst reduction was associated with lower salty flavor and less think texture. However, as with satiety expectations no study to date has explored whether expression of these thirst-based expectations depends on acute thirst. The same theoretical argument can be made here as with satiety: if thirst-related expectations are based on recall from memory of past experience with a particular product or group of products, it seems unlikely that acute thirst would modify those expectations. But if acute thirst promotes attention to features of the product relevant to its potential thirst-quenching ability, then actual thirst could be predicted to magnify product-related expectations of thirst reduction.

In designing a study to test the possible effects of acute hunger and thirst on evaluations of expectations of satiety and thirst reduction for different products we were aware that one theoretical perspective predicted no effect of motivational state. A concern with a null-result finding, however, would be that this reflected inadequate test sensitivity rather than a lack of effect of need state. To counteract this, we incorporated two manipulations that should result in measurable differences in satiety and thirst-related expectations independent of current appetite. Firstly, we chose two product classes: two soups and two beverages. Past research consistently shows that soups in general are expected to be more satiating than are drinks (Himaya and Louis-Sylvestre, 1998; Mattes, 2005), and conversely we predicted that drinks would be predicted to reduce thirst more than would soups. Secondly, we used a more subtle manipulation based on the thickness of the tested products, since thicker texture has previously been shown to moderate expectations of satiety and thirst (McCrickerd et al., 2012; Chambers et al., 2015). We therefore tested two versions of each type of product (soup and beverage), one with a thicker texture and one thinner, with the clear prediction that thicker products would be predicted to be more satiating and less thirst-quenching than thinner products. These two manipulations provided control effect sizes against which we could contrast any effects of the key manipulation of acute hunger and thirst. The final study design therefore contrasted the effects of manipulated thirst and hunger on satiety and thirst-related expectations of four test products, using a between participants design with three motivational-state groups: hungry, thirst and sated.

## Materials and Methods

### Design

Evaluations of expected satiation, satiety, thirst reduction and thirst suppression were made for thinner and thicker examples of two types of products (drinks and soups) 2 h after a breakfast session where healthy volunteers either had *ad libitum* access to food and drink (Sated condition), access to beverages only (Hungry condition) or access to food but no beverages (Thirsty condition), using a between participants design.

### Participants

Seventy two healthy volunteers (36 men and women) were recruited, mainly from staff and students at the University of Sussex. Potential participants were told that the study examined the effects of breakfast on mood and performance to disguise the interest in expectations. Potential participants aged outside the study range (18–55 years), with allergies or aversions to any of the products or ingredients being used, who were diabetic, who had a prior diagnosis of an eating disorder, who smoked more than five cigarettes a week, who were taking a prescribed medicine or who were pregnant or breast-feeding were excluded. All potential participants completed the Three Factor Eating Questionnaire (TFEQ: Stunkard and Messick, 1985) as part of the recruitment process, and those scoring seven or more on the TFEQ Restraint scale were also excluded to reduce the possibility of evaluations of the products under test being affected by having a restrained eating attitude. To control for gender differences in responses equal numbers of men and women were tested in each of the three conditions. The study was carried out in accordance with the recommendations of the British Psychological Society Code of Ethics, with written informed consent from all participants in accordance with the Declaration of Helsinki. The protocol was approved by the Science and Technology Cross-Schools Research Ethics Committee of the University of Sussex.

### Test Food and Drinks

Participants attended two sessions 2 h apart: a breakfast session designed to manipulate motivational state, and a mid-morning taste test where the expectation data were collected.

#### Breakfast Session

Breakfast was served to groups of participants as an *ad libitum* buffet. The key difference between the three test conditions was which food and drink items were available. The beverages used in this study were: reduced energy cranberry juice (Ocean Spray Cranberry Classic Light: 16 kcal/100 ml), orange juice (Sainsbury’s brand: 42 kcal/100 g), semi-skimmed milk (Sainsbury’s brand: 50 kcal/100 g), full-cream Jersey milk (Thirst condition only: Sainsbury’s brand; 79 kcal/100 g) and mineral water. The food used was a standard breakfast cereal (Crunchy Nut Cornflakes, Kellogg’s brand, 119 kcal/100 g). In the Sated (control) condition, all of these items (except the full-cream milk) were freely available from a self-serve buffet, while in the Hunger condition only the water and cranberry juice were available and in the Thirsty condition only the cereal and full-cream milk were available. Participants reported for breakfast at either 0830 or 0900 h, and had a maximum of 30 min access to the buffet. Only one condition could be served at each slot, and the three test conditions were randomly assigned across test days and time slots to minimize spurious effects of day and time of testing.

#### Test Products

Pilot tests, where we contrasted rated expectations of satiety and thirst reduction across a range of commercial products, were used to select four items that consistently rated as thinner and thicker versions within each of the two product categories being tested: beverages and soups. The product classified as the thinner-textured beverage was “Coconut-water, pineapple and passion fruit juice drink” (Sainsbury’s, United Kingdom: 12 kcal/100 ml), while the thicker beverage was “Pineapple, banana and coconut smoothie” (Innocent brand, Coca-Cola United Kingdom: 66 kcal/100 ml). The thinner soup was a commercial packet-soup (“Cup-a-soup Golden Vegetables,” Batchelors, United Kingdom: 32 kcal/100 g) prepared following manufacturer’s instructions, while the thicker soup was “Tomato, Lentil and Red Pepper soup” (Sainsbury’s United Kingdom: 65 kcal/100 g). Both beverages were served below room temperature (at 10–15°C), and the soups warmed in a microwave (temperature when provided to participants between 60 and 75°C).

### Procedure

After completion of the recruitment procedures, participants attended for two separate sessions (breakfast and mind-morning test) on a single test day, having been instructed to eat nothing and to drink water only from 2300 h on the previous evening. The breakfast session was held in a large room separate from the main testing laboratory. After completing the consent procedures, participants were told that they were welcome to consume as much as they liked of the available products. Which products were available then depended on the test condition. In the Sated condition, all breakfast products were available, and participants were allowed to consume as much as they liked of all products. Once they had finished, they were simply given instructions on the time and location of the second test session and were not given any instructions to restrict their intake in the intervening 2 h. In the Hungry condition, the only products on offer were the low-energy cranberry juice and water, and once they had consumed all they wanted they were instructed that they could drink water only, but were not allowed to eat anything, before the next test session. Finally, those in the Thirsty condition only had access to the cereal and milk for breakfast and were instructed that they were welcome to eat anything that they liked but were not allowed to drink anything more before the test session.

The procedure at the second session was the same for all participants. Initially, participants completed a standard battery of ratings of mood and appetite ratings. These were disguised as “Mood Questions” to corroborate the study’s cover story (“investigating the effects of breakfast on mood and performance”). Participants were asked “How <mood/appetite descriptor> do you feel right now?” and were instructed to respond by placing a marker along a 100 point visual analog scale (VAS) positioned in the middle of the screen end-anchored “Not at all <descriptor>” (0) to “Extremely <descriptor>” (100). The descriptors of appetite state evaluated how hungry and thirsty they felt, and these ratings were mixed at random with a range of mood related items (tired, happy, headachy, anxious, nauseous, energetic, and alert). Ratings were presented in randomized order and only the appetite ratings were analyzed. Ratings were presented using a customized program generated using MatLab (version R20112b) running on Dell Windows PC computers.

On completion of the mood/appetite ratings, participants were prompted to call their experimenter, who provided them with a tray containing 20 g samples (served in paper cups with 28 ml serving volume) of the four test products along with a large glass of water. The testing protocol was again presented on a PC computer using Matlab, and the program instructed the participant to select one product (labeled using 3 digit codes), to taste that product, and then to complete a number of evaluations about that product. To standardize sampling time, participants were instructed to keep the sample in their mouth for 3 s and then to swallow that sample, in line with procedures we have used before (McCrickerd et al., 2012). The order in which the samples were tested was randomized. First, participants rated the sensory characteristics of the tasted sample, presented as computeried VAS end-anchored in the same format as the earlier mood/appetite ratings. The dimensions rated were for how pleasant, sweet, salty, thick, creamy and familiar the product tasted. These data allowed us to confirm the two control manipulations (product type and product sensory characteristics), as well as to test how these were modified by acute appetite state. Secondly, the four key expectation measures of what effect consuming these products were expected to have on appetite and thirst were presented, using the same measures as those used in previous studies in our laboratory (McCrickerd et al., 2015). In brief, participants were provided with two reference stimuli, a glass and a clear glass bowl both containing 300 ml of blue colored liquid as a portion guide and were asked to consider their expectations for the impact of consuming that portion of the tested beverage or soup using the instruction “Imagine you have just consumed a WHOLE SERVING of the product.” Expected satiation or thirst quenching were assessed by the question “How FULL/THIRSTY would you feel IMMEDIATELY afterwards?”, while expected satiety/thirst suppression using the wording “How HUNGRY/THIRSTY would you feel in 1 h time?”. All ratings again were made using 100pt VAS in the same format as the earlier mood/appetite ratings.

We also tested the expected utility of each of these products by asking them the question “How much would you be willing to pay for a whole serving of this product?” and they entered this value as a number (in United Kingdom pence) into a response box on the computer screen, again using the colored liquid portions as a reference guide. After testing each product, participants were asked to rinse their mouth with water before testing the next product. Once all four products had been evaluated, participants age, height and weight were recorded, and they were debriefed about the true purpose of the study.

### Data Analysis

One male participant declined to provide age, height and weight data, but all other data were complete. To test for spurious differences between groups tested in the three conditions, age and BMI were contrasted by group and gender using 2-way ANOVA. The key focus was on the immediate and delayed satiety/thirst expectations. Initial analyses included gender in these analyses but the only significant effects involving gender were a weak 4-way interaction for expected satiety with small effect size [*F*(2,66) = 4.53, *p* = 0.01, η^2^= 0.12], and a weak 3 way interaction not involving condition for expected satiation [*F*(1,66) = 4.95, *p* = 0.03, η^2^ = 0.07]. Given the large number of contrasts made, we had not hypothesized any effects of gender, and inclusion of gender reduced study power, the reported outcomes do not include gender in these analyses, although we note that reported significant effects remained significant when gender was included. Each of the four key expectation ratings were contrasted between the test condition (Hungry/Thirsty/Sated) between participants and two control manipulations (product type and sensory characteristics: both within participants) using mixed ANOVA. The same analysis was also used to explore effects of the motivation manipulation on actual sensory ratings, and the value for utility (in pence). Hunger and thirst at the start of the test session were contrasted by group to verify the success of the breakfast manipulation. The analyzed dataset can be accessed at 10.25377/sussex.7334480.

## Results

### Group Characteristics

Participants in the three test conditions (Table 1) did not differ significantly in age [*F*(2,65) = 0.83, *p* = 0.44, η^2^= 0.01] or BMI [*F*(2,64) = 0.32, *p* = 0.72, η^2^= 0.01], nor were there any significant differences in age or BMI between the women and men tested in these groups.

**Table 1:** Characteristics of the participants in the three test conditions.

	Test condition		
	Hungry	Thirsty	Sated
Age (years)	22.3 ± 0.7	21.1 ± 0.8	22.2 ± 0.5
BMI (kg/m^2^)	21.9 ± 0.6	21.6 ± 0.6	22.2 ± 0.4


*All data are mean ± SEM*.

### Rated Hunger and Thirst Prior to the Taste Test

Analysis of the hunger and thirst ratings made at the start of the taste test confirmed the success of the breakfast manipulation (Figure 3). Hunger differed significantly between conditions [*F*(2,66) = 22.24, *p* < 0.001, η^2^= 0.40], with participants in the Hungry condition significantly more hungry than those in the Thirsty or Sated conditions. Likewise, thirst differed significantly between conditions [*F*(2,66) = 10.79, *p* < 0.001, η^2^= 0.25], with participants in the Thirsty condition significantly more thirsty than those in the Hungry or Sated conditions.

### Expectations About Appetite and Thirst

**FIGURE 1:**
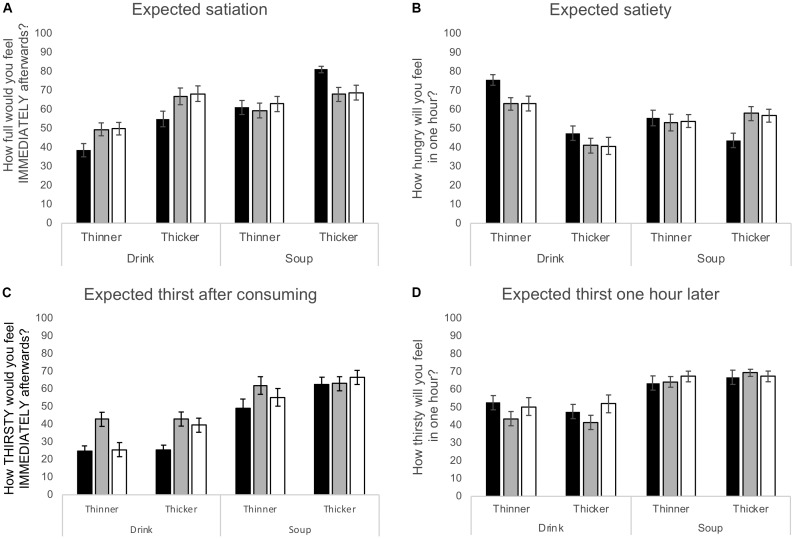
Ratings of **(A)** expected satiation, **(B)** expected satiety, **(C)** expected thirst reduction and **(D**) thirst suppression for the four test products evaluated by participants in the Hungry (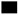), Thirsty (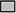), and Sated (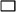) test conditions. All data are mean ± SEM.

As predicted, expected satiation (i.e., how full people expected to be after consuming each product) depended both on the type of product (drink or soup: [*F*(1,69) = 43.30, *p* < 0.001, η^2^= 0.39]) and whether the version rated was thinner or thicker in texture [*F*(1,66) = 74.24, *p* < 0.001, η^2^= 0.52]. However, although these ratings did not differ between conditions overall [*F*(2,69) = 0.50, *p* = 0.61, η^2^= 0.02], there was a significant interaction between condition and product type [*F*(2,69) = 10.76, *p* < 0.001, η^2^= 0.24]. On average soups were rated as being expected to be more filling than drinks (Figure 1A), however, both drinks were expected to be less filling in the Hungry than Thirsty or Sated conditions, while soups were expected to be more filling when hungry, particularly driven by the thick soup. Thus, hunger state magnified the difference in expected satiation between drinks and soups. The same effect was not seen in relation to the sensory manipulation (condition × sensory interaction [*F*(2,69) = 1.28, *p* = 0.29, η^2^ = 0.04]: condition × sensory × product interaction [*F*(2,69) = 2.05, *p* = 0.14, η^2^= 0.06]).

Expected satiety (how hungry people expected to feel an hour after consuming these products: Figure 1B) also varied with the product sensory characteristic overall [*F*(1,69) = 42.77, *p* < 0.001, η^2^= 0.38], with lower hunger predicted after the thicker than thinner products, but surprisingly there was no main effect of product type [*F*(1,69) = 0.81, *p* = 0.37, η^2^= 0.01]. However, there were significant interactions between condition and product type [*F*(2,69) = 7.83, *p* < 0.001, η^2^= 0.19], between sensory and condition [*F*(2,69) = 3.40, *p* = 0.04, η^2^= 0.09], and sensory and product type [*F*(1,69) = 24.05, *p* < 0.001, η^2^= 0.26]. Overall, expected delayed hunger was surprisingly similar for the two soups (thinner soup 54 ± 2, thicker soup 53 ± 2), but differed between the thinner (67 ± 2) and thicker (43 ± 2) drinks. However, whereas the pattern of differences in expected satiety across products was almost identical in the Thirsty and Sated groups, those in the Hungry group expected to be more hungry 1 h after consuming drinks, and less hungry after consuming soups, than were the other two groups. Likewise, they expected to be less hungry after the thicker, and more hungry after the thinner products, than the other groups.

Expectations about how thirsty people expected to be immediately after consuming each product (Figure 1C) varied depending on whether this was a drink or soup [*F*(1,69) = 163.64, *p* < 0.001, η^2^= 0.70], with lower expected thirst after drinks than soups as expected, and also depending on the sensory manipulation [*F*(1,69) = 13.84, *p* < 0.001, η^2^= 0.17], with lower expected thirst after thinner than thicker versions. These ratings also differed overall between conditions [*F*(2,69) = 6.39, *p* = 0.003, η^2^= 0.16], with people expecting to be significantly thirstier after consuming any of the products in the Thirsty than the Hungry condition, with the Sated condition intermediate. There was also a significant interaction between sensory and condition [*F*(2,69) = 3.71, *p* = 0.03, η^2^= 0.10]: here, expected thirst was predicted to be the same after the thin and thick versions in the Thirsty condition, but to be less after the thin than thicker versions in the Hungry and Sated conditions, where these ratings were lower overall.

Ratings of how thirsty people expected to be an hour later (Figure 1D) only differed significantly with product type: as expected, people expected to be thirstier 1 h after consuming soups than drinks [*F*(1,69) = 661.5, *p* < 0.001, η^2^= 0.49], with no significant effects of sensory [*F*(1,69) = 0.11, *p* = 0.74, η^2^= 0.01], condition [*F*(2,69) = 0.75, *p* = 0.47, η^2^= 0.02], and no significant interactions.

The wording of the expectation questions all asked participants to predict their hunger or thirst after consuming each product. As we deliberately altered hunger and/or thirst at the time these ratings were made, apparent differences in expectations between groups could simply reflect actual baseline differences. To test this we re-evaluated the differences in he expectations between groups and products while adjusting for baseline appetite. As we did not have baseline ratings of fullness, we used an approximation of baseline fullness as the inverse of baseline hunger (calculated as 100 – baseline hunger). Although not ideal, this approach is supported by the widespread observation that of hunger and fullness are strongly correlated (Booth, 1987; Yeomans and Bertenshaw, 2008; Blundell et al., 2010; Yeomans, 2018), and by the recommendation to integrate ratings of fullness and hunger into a single measure of satiety (Blundell et al., 2010). For expected satiation, the interaction between product type and condition remained significant after adjusting for baseline fullness [*F*(2,69) = 10.76, *p* < 0.001, η^2^= 0.24], with the thick soup still predicted to increase fullness more, and the think drink less, when hungry than in the other conditions. Likewise, the interactions between product type and condition [*F(*2,69) = 6.62, *p* = 0.002, η^2^= 0.16] and sensory and condition [*F*(2,69) = 3.18, *p* = 0.04, η^2^= 0.08] remained significant when predicted hunger in 1 h was adjusted by baseline hunger.

For thirst, again the differences in baseline thirst could not explain the product-specific differences in immediate thirst reduction between conditions: thus, a significant interaction between sensory and conditions [*F*(2,69) = 3.71, *p* = 0.03, η^2^= 0.10] remained after adjusting for actual thirst. However, predictions of how thirsty people would be an hour after consuming the different products was unaffected by condition when adjusted by baseline thirst.

### Rated Sensory and Hedonic Characteristics

Rated liking (Table 2) was similar for the four test products, and these ratings did not differ between test conditions (main effect of condition: [*F*(2,69) = 0.06, *p* = 0.94, η^2^= 0.01]): interactions between condition and product type or sensory characteristics were non-significant. Likewise, the four test products were similar in familiarity, which did not differ significantly between conditions. Sensory ratings (Table 2) were analyzed to confirm that the four products differed as predicted. As expected, soups were rated as thicker than drinks overall [*F*(1,69) = 157.52, *p* < 0.001, η^2^= 0.70], and the items pre-selected as “thicker” examples were perceived as thicker [*F*(1,69) = 521.05, *p* < 0.001, η^2^= 0.88]: there was also a significant food-type × sensory interaction for thickness ratings [*F*(1,69) = 21.38, *p* < 0.001, η^2^= 0.24], with the thinner drink rated much less thick than the thinner soup. There was no significant overall effect of condition [*F*(2,69) = 0.28, *p* = 0.76, η^2^= 0.01], nor any significant interaction involving condition, on rated product thickness. Rated creaminess followed the same pattern as thickness: soups were overall rated more creamy than drinks [*F*(1,69) = 104.96, *p* < 0.001, η^2^= 0.60], and the two thicker items more creamy than the thinner ones [*F*(1,69) = 120.28, *p* < 0.001, η^2^= 0.64] with a significant interaction [*F*(1,69) = 58.75, *p* < 0.001, η^2^= 0.46] due to very low creamy ratings for the thin drink. Motivational state had no significant effects on creaminess perception. As would be expected, the two drinks were rated as sweeter than the soups [*F*(1,69) = 189.37, *p* < 0.001, η^2^= 0.73], but sweetness was not affected significantly by motivational state or the sensory manipulation. Ratings of how salty the products were, however, did depend on condition (significant interaction between condition × food type × sensory: [*F*(2,69) = 5.04, *p* = 0.009, η^2^= 0.13], as well as depending on food type [*F*(1,69) = 387.57, *p* < 0.001, η^2^= 0.85], sensory [*F*(1,69) = 5.64, *p* = 0.02, η^2^= 0.08] and the interaction of sensory and food type [*F*(1,69) = 19.24, *p* < 0.001, η^2^= 0.22]). Overall, soups were rated saltier than drinks, as expected, and thinner soup was the saltiest. However, those in the Thirsty group rated the thick soup as less salty than did those in the Hungry and Sated conditions (Table 2).

**Table 2:** Rated characteristics of the four test products in the three motivational conditions.

Rating	Drink						Soup					
	Thinner			Thicker			Thinner			Thicker		
	Hungry	Thirsty	Sated	Hungry	Thirsty	Sated	Hungry	Thirsty	Sated	Hungry	Thirsty	Sated
Pleasant	64 ± 4	64 ± 4	68 ± 4	59 ± 6	66 ± 5	65 ± 5	66 ± 5	67 ± 4	57 ± 5	65 ± 3	62 ± 4	62 ± 3
Familiar	75 ± 4	70 ± 4	72 ± 5	71 ± 3	72 ± 4	71 ± 4	70 ± 3	69 ± 3	61 ± 4	70 ± 4	64 ± 4	71 ± 2
Thick	16 ± 2	18 ± 4	13 ± 2	64 ± 5	62 ± 4	67 ± 3	41 ± 3	43 ± 4	49 ± 4	78 ± 2	78 ± 2	79 ± 2
Creamy	13 ± 3	16 ± 3	13 ± 2	55 ± 5	59 ± 5	53 ± 5	59 ± 5	51 ± 4	54 ± 5	69 ± 3	64 ± 3	54 ± 4
Sweet	75 ± 4	69 ± 3	79 ± 2	73 ± 4	73 ± 4	78 ± 2	42 ± 4	41 ± 5	32 ± 4	36 ± 5	36 ± 5	34 ± 4
Salty	14 ± 3	13 ± 2	12 ± 2	16 ± 3	17 ± 4	13 ± 2	61 ± 3	59 ± 3	73 ± 3	61 ± 3	45 ± 5	65 ± 2


*Data are mean ± SEM visual analog ratings on 100 pt scales*.

### Product Utility Measure

**FIGURE 2:**
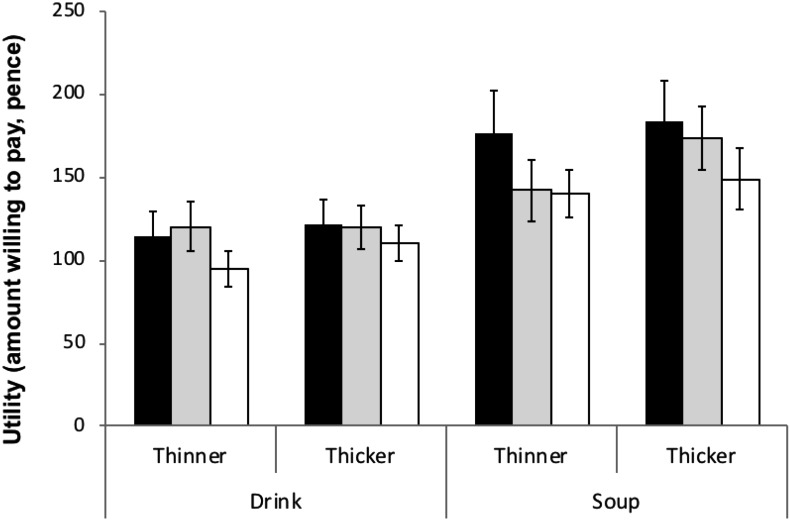
The amount people were willing to pay for the four test products in the Hungry (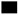), Thirsty (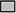), and Sated (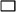) test conditions. All data are mean ± SEM.

Overall participants were willing to pay more (Figure 2) for soups than drinks [*F*(1,69) = 33.58, *p* < 0.001, η^2^= 0.33], and for thicker than thinner versions of each product type [*F*(1,69) = 4.37, *p* = 0.04, η^2^= 0.06], but these evaluations were unaffected by motivational state (main effect of condition: [*F*(2,69) = 0.88, *p* = 0.42, η^2^= 0.03]: no significant interactions between condition, sensory and product type).

**FIGURE 3:**
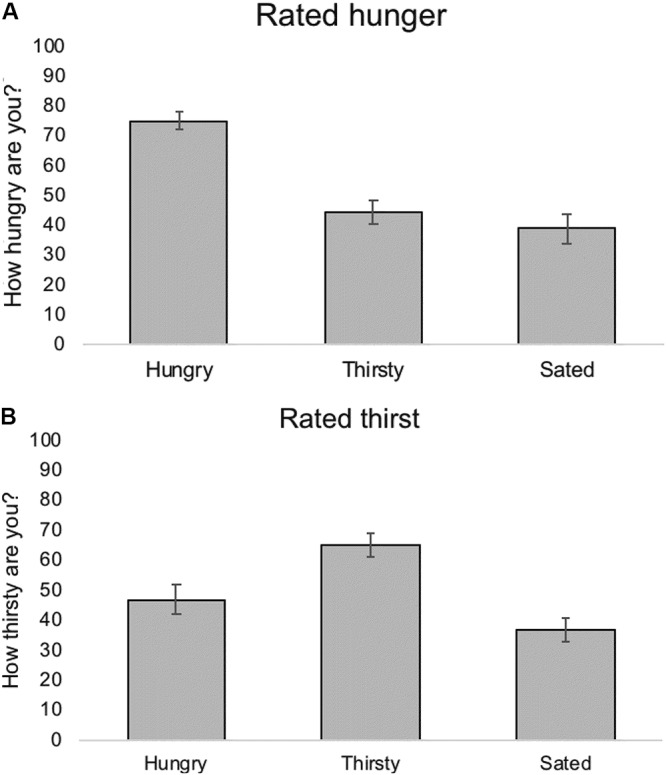
Rated **(A)** hunger and **(B)** thirst for participants in the three test conditions. All data are mean ± SEM.

## Discussion

The current study is the first to provide clear evidence that when people make judgments about the likely impact of ingesting different foods and drinks on their appetite and thirst, these expectations are acutely but subtly adjusted by the person’s current appetitive state. This is an important finding since it suggests that we have mechanisms that highlight the most important features of a product at the time when it may be most beneficial to the consumer: accordingly, the product that be most effective in increasing fullness (here a thicker-textured soup) was predicted to be more filling when consumed by the hungrier group, whereas the product which would have the least effect (a thin-textured drink) was expected to be even less effective at reducing hunger when evaluated in a hungry state.

One of the most widely cited concepts in current theories of motivation is incentive salience (Berridge and Robinson, 2003; Berridge, 2004; Smith et al., 2011), which is usually defined in terms of the extent to which attention to an object motivates approach to that object, and is part of a broader concept of motivational salience, which encompasses both approach and avoidance of motivationally relevant objects (Bromberg-Martin et al., 2010; Cunningham and Brosch, 2012). These concepts maybe useful in explaining our findings: here, the higher appetitive state of the consumers in the hungry condition may increase attention to those features of the products on test which would be most valuable to the consumer at that time: i.e., the incentive salience of those products which were most “food-like” was enhanced by hunger. The different motivational states did not alter the overall qualitative differences in the way the four test products were predicted to alter appetite and thirst: in all cases the thick soup was expected to be the most filling and the thin drink the least. But what motivation state appeared to do was magnify those differences, in effect drawing the consumers attention to the product or product features that would have greatest benefit in their current motivational context. To date, studies on incentive salience have tended to focus on attention to food cues in relation to acute hunger (e.g., Castellanos et al., 2009; Siep et al., 2009), without considering the way these attentional processes might be used to detect subtle differences in product utility. Our data suggest that a more nuanced approach is needed to uncover relative attention to different features of a product.

Further implicit support for an incentive salience interpretation of our current finding was the lack of difference in liking for the different products across the different motivational conditions. The four products used in this study were generally liked, and there were no clear differences in liking between products. Crucially, liking did not vary with motivational state, unlike measures of expected satiation and satiety. Theories of motivation that suggest a dissociation between liking and motivational-value (wanting) have likewise suggested that wanting not liking increases with deprivation (e.g., Epstein et al., 2003; Finlayson et al., 2009), and our data fit with that analysis. Recent research also suggest that product choice integrates liking with expected satiety (Brunstrom and Shakeshaft, 2009; Brunstrom et al., 2016). But those studies assumed expected satiety to be independent of appetite state, whereas our data go one step further and suggest that as our appetite increases, so our attention is more drawn to those features of a product that predict the most beneficial effects on our hunger state. Thus our data suggest that models of product choice must critically incorporate acute appetitive state as a key factor in determining relative attention to different aspects of the likely benefits of consumption.

The current study is also one of the few to so far explore expectations about thirst reduction alongside expected satiety. Whereas the data on expected satiation and satiety showed clear effects of motivational state on the evaluation of the four products that could not be explained simply by the baseline differences in hunger, there was less evidence that expectations of thirst were similarly modified by acute thirst. There was some evidence that drinks were predicted to be better at reducing acute thirst when acutely thirsty, whereas soups were expected to have the same effect on thirst regardless of acute appetite, but this difference did not extend to expectations of thirst 1 h after consumption. Since both satiety and thirst expectations are believed to result from memory of prior experience of consumption (Brunstrom et al., 2012), it may be that the effects of products on thirst are much more consistent and less variable across products than are effects on satiety. Likewise, the subtle difference between some effects of motivational state on immediate but not delayed thirst may reflect a greater influence of oral factors in immediate thirst reduction (Brunstrom et al., 2000; Labbe et al., 2009) but actual likely effects on rehydration for delayed thirst expectations.

In order to provide evidence of sensitivity of the test measures, we contrasted two types of product and two levels of sensory characteristics (thinner or thicker texture). The clear differences in all expectation measures between soups and drinks, and thicker and thinner versions, suggests that our measure was sensitive and are in line with the broader literature pointing to a products’ sensory characteristics and consumption context as important cues guiding its expected and experienced satiating power (McCrickerd and Forde, 2016). In particular, previous studies have suggested that the same products consumed in different contexts (e.g., ‘drink’ vs. ‘food,’ or ‘meal’ vs. ‘snack’) can differentially impact appetite (Mattes, 2005; Capaldi et al., 2006; McCrickerd et al., 2014), whereas equicaloric foods and beverages are often expected to be more satiating when they are presented with thicker, more viscous or harder and chewier sensory characteristics (Forde et al., 2013, 2017). We chose to use ratings of expected satiety rather than the more psychophysical measures widely used by Brunstrom and colleagues (see Brunstrom, 2011) since in a recent study where we included both rating and psychophysical measures of expected satiety (based on matching visual images of different portion sizes on equivalent expected effects on appetite), we found evidence that the rating measure adjusted to actual nutrient content with repeated consumption but the psychophysical measure did not (Yeomans et al., 2014), suggesting that ratings may be more sensitive to subtle state manipulations when making judgments of tasted food products.

Another critical aspect of the present design was to provide evidence that the expected differences in sensory characteristics of the four test products were evident to the actual participants, and there was clear evidence that this was so. Thus for both the drinks and soups, the products pre-selected to be thicker were consistently rated as thicker and creamier than were the versions of these products selected to be thinner. Notably there were no clear differences in familiarity between products, which was important since there is evidence that measures of expected satiety become more attuned to actual nutrient content with familiarity (Brunstrom et al., 2010). As we used a between-groups design, there was also some risk that spurious differences in sensory evaluation and familiarity with these products between groups might have confounded any differences in the expectation measures, but there was no evidence of any difference between groups on any of the sensory, familiarity or liking measures.

Because of concerns about the potential demand effects that could have arisen if people had completed all three test sessions in a within-participants design, between-groups contrasts were used to assess the effects of hunger and thirst, using the two control manipulations to test for potential spurious group differences in the key expectation measures. Future studies should seek to replicate these findings using a within-participants design perhaps using disguised energy preloads to manipulate hunger state to ensure the reported effects were not due to unexpected group differences. The present study also focussed exclusively on liquid and semi-liquid products because of our dual interest in hunger and thirst expectations: the state-dependent effects of expected satiety should also be explored using other types of products, including solid foods, and a number of other sensory manipulations to better understand the cues most relevant to consumers varying in motivational state.

In summary, the present study provide the first clear evidence that acute motivational state subtly alters the extent to which consumers are aware of differences in the likely impact of ingestion on their appetite, with the data suggesting that acute hunger exaggerates differences in the extent to which products are expected to appease hunger and to a lesser extent thirst.

## Author Contributions

MY, LC, and KM contributed equally to study design and data collection. MY took the lead in the reported data analysis and drafting of the MS. KM and LC commented on draft versions.

## Conflict of Interest Statement

The authors declare that the research was conducted in the absence of any commercial or financial relationships that could be construed as a potential conflict of interest.
